# Social relationships promote access to food and information in wild jackdaws

**DOI:** 10.1098/rspb.2026.1462

**Published:** 2026-10-07

**Authors:** Luca G. Hahn, Guillam E. McIvor, Ines Fürtbauer, Andrew J. King, Alex Thornton

**Affiliations:** ^1^ Centre for Ecology and Conservation, University of Exeter, Penryn, Cornwall TR10 9FE, UK; ^2^ Biosciences, Faculty of Science and Engineering, Swansea University, Singleton Park, Swansea SA2 8PP, UK; ^3^ Department of Behaviour and Cognition, University of Vienna, Vienna 1030, Austria

**Keywords:** social foraging, social learning, social relationships, social tolerance

## Abstract

Many animals form differentiated social relationships that can influence fitness, but the mechanisms behind these benefits are often unclear. How does the presence of social partners during foraging enhance access to food or information? Might individuals also incur short-term costs, for example, by foregoing feeding opportunities to remain with and support social partners? Here, we investigated how social relationships shape foraging decisions and outcomes in wild jackdaws (*Corvus monedula*) using automated radio frequency identification (RFID) feeding stations where two birds could perch but only one could feed, with the other queueing behind (‘dyadic events’). Bonded social partners (pair-bonded mates and kin) engaged in these dyadic events more frequently than other dyads and they coordinated their visits, as shown by shorter dyadic arrival latencies. Bonded partners also showed higher social tolerance and possibly social support, with feeding individuals benefitting from a lower probability of being displaced when a partner was present. Queuing individuals accepted short-term time costs to remain with their social partners. For juveniles, following their parents probably promoted access to social information about feeder use. These findings show that social relationships influence decision-making and foraging performance in the wild, revealing both benefits and costs of maintaining close bonds. Short-term effects on foraging success may therefore contribute to long-term fitness advantages of strong social relationships.

## 1. Introduction

Many animals form and maintain differentiated social relationships among both kin and non-kin [1,2]. Such relationships, typically defined as repeated affiliative social interactions between specific individuals [1,3], result in structured social networks [4–6] and may provide important benefits. For instance, correlational studies on ungulates [7,8], primates [9,10] and cetaceans [11,12] indicate that individuals with stronger and/or more social relationships tend to be healthier, have higher reproductive success and live longer [13–16]. However, the mechanisms underlying such benefits are often unclear [17,18]. To understand how these benefits (and associated costs) arise, we must investigate the immediate consequences of individuals' decisions about whom to associate with and how to behave towards others. For instance, the short-term consequences of individuals' decisions when foraging with others could provide a mechanism through which long-term benefits of social relationships emerge.

When foraging, animals may benefit from preferentially associating with bonded partners, such as pair-bonded mates, kin or ‘friends’. Individuals choose to forage alongside bonded partners in various species, including dwarf mongooses (*Helogale parvula*) [19], vampire bats (*Desmodus rotundus*) [20], Assamese macaques (*Macaca assamensis*) [21] and chacma baboons (*Papio ursinus*) [22]. To achieve foraging benefits, bonded partners may need to coordinate their activities and show social tolerance (i.e. proximity without aggressive behaviour). This requires the use of social information and decision rules to discriminate among individuals: for example, domestic dogs (*Canis lupus familiaris*), wolves (*Canis lupus*) [23] and vervet monkeys (*Chlorocebus pygerhythrus*) [24] show higher social tolerance towards bonded partners than other individuals. Social tolerance and support between bonded partners may also enable better access to contested resources and social information, potentially resulting in increased immediate food intake [25] and social learning [26–28], which could be particularly important for younger, naive individuals. For instance, in common ravens (*Corvus corax*) [29], Barbary macaques (*Macaca sylvanus*) [30] and bearded capuchins (*Sapajus libidinosus*) [31], proximity to individuals solving experimental extractive foraging tasks, requiring social tolerance, predicted social learning by observers.

To understand the role of social relationships and social tolerance during foraging, we must consider not only the benefits, but also the costs. For example, individuals may trade off longer-term benefits of maintaining group cohesion (e.g. reducing predation risk) with shorter-term costs (e.g. reduced foraging success). Experiments with chacma baboons provide evidence for this: lower-ranking baboons follow the movement decisions of higher-ranked individuals to food patches where they will receive less food but remain as a cohesive troop [32]. In other cases, costs may be linked to specific dyadic social bonds. Great tits (*Parus major*), for example, reduce their own foraging payoffs to remain near their foraging pair-bonded partner [33]. This could reflect obligate biparental care, as individuals may be willing to incur short-term costs to ensure that their partner has sufficient resources to contribute to rearing offspring. This might be especially important for genetically monogamous species, where the fitness outcomes of both partners are highly interdependent, but this has yet to be examined.

An ideal system to investigate social decision-making during foraging is the jackdaw (*Corvus monedula*), a highly social member of the Corvidae family. Jackdaw society is centred around long-term pair bonds, which can last a lifetime and are reported to be genetically monogamous [34,35]. Pair members coordinate their activities throughout the year [36] and across contexts, including nest building [37], incubation [38], flocking [39] and foraging [40]. Relationships between kin, particularly between parents and their offspring, are also important. Juveniles remain dependent on, and retain close associations with, their parents as they transition to independence in the weeks and months after fledging [41]. These strongly bonded partners are embedded in a dynamic social network with fluid membership [42], so individuals regularly interact with many others with whom they share varying levels of familiarity and relationship strength. For example, as colony-breeding cavity nesters that readily use nest boxes, jackdaws may be particularly familiar with neighbours whose nests are nearby. Therefore, while individuals may form enduring social associations with familiar individuals that are not pair-bonded mates or kin (i.e. ‘friends’ [2]), many interactions with less familiar individuals may be characterized by competition and limited social tolerance. Finally, jackdaws are generalist foragers that regularly forage socially [41], at monopolizable food sources [41], which is facilitated by social information use [43,44].

We tested the overarching hypothesis that jackdaws' social relationships shape decisions and encounters during foraging, with consequences for accessing food and information. To this end, we recorded dyadic encounters at radio frequency identification (RFID) feeding stations, where one jackdaw could feed at a time, while another could queue behind at a second perch in close proximity (hereafter referred to as ‘dyadic events’). We compared strong relationships (bonded partners: pair bonds and kin) with other, more transient relationships with varying levels of familiarity (breeding neighbours, foraging associates, same-site residents and residents from different study sites) and tested several linked predictions: (i) *Social preference.* Bonded partners should visit dyadic feeding stations together more frequently compared with other dyads. Neighbours and foraging associates may also visit together more frequently than same-site residents and residents from different sites. (ii) *Coordination.* We predicted that bonded partners should arrive at feeding stations closer in time than other dyads. (iii) *Social tolerance.* We predicted that (a) bonded partners should queue for longer compared with other dyads (i.e. show greater social tolerance). Neighbours and foraging associates may queue for longer compared with same-site residents and residents from different sites. (b) Individuals at the primary feeder perch (i.e. the perch with access to food) should be less likely to be displaced by a third party if an adult bonded partner (pair-bonded mate or parent) is queuing behind them compared with when another adult or no other bird is queueing behind. This could potentially reflect social support, improving individuals' access to food. (c) Individuals at the primary feeder perch could more readily allow bonded partners to feed next compared with other birds queuing behind them. Conversely, individuals queuing behind their bonded partners could also accept the short-term cost of foregoing feeding. (iv) *Social learning opportunities.* We predicted that if juveniles followed their knowledgeable parents (i.e. those who used feeding stations) during their first visit to a focal feeding station, they would visit that feeding station (a) earlier and (b) more often than if their first visit was not with parents.

## 2. Methods

### (a) Study system

We studied jackdaws at two field sites centred around two nest-box colonies in Cornwall, United Kingdom: site Y, in a churchyard and surrounding fields in the village of Stithians (50°11′26″ N, 5°10′51″ W) and site Z, a farm approximately 1 km away (50°11′56″ N, 5°10′9″ W) (figure 1a ). Jackdaws at the study sites are colour-ringed for individual identification, and one of the colour rings contains a uniquely identifiable RFID tag (*Eccel Technology*, formerly *IB Technology*, UK). Pair bonds, kin relationships and other relationship types were determined through long-term monitoring of sightings, nest-box occupancy, breeding attempts and data from RFID feeding stations.

**Figure 1.**
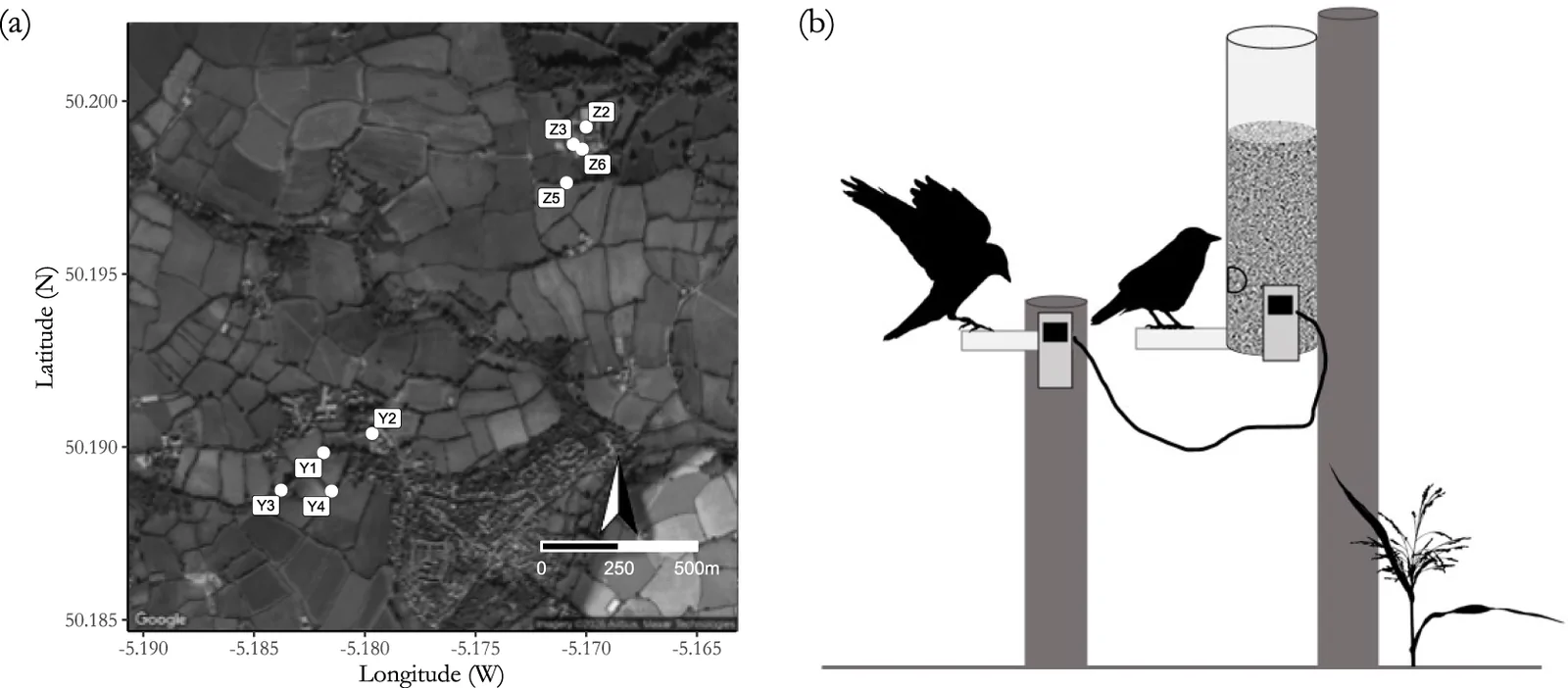
(a) Locations of dyadic feeding stations fitted with two RFID perches at the two study sites Y and Z in 2023 and 2024. The dyadic feeding station at location Z3 in 2023 was moved to location Z6 in 2024, and the feeding station Y4 was not used in 2024. The map was created using the packages *ggmap* (v. 4.0.0) [45], *ggsn* (v. 0.5.3) [46] and *ggrepel* (v. 0.9.6) [47]. (b) Set-up of dyadic feeding stations with primary (feeder) and secondary perches (note that the RFID feeding station and logger design are simplified representations). At single-perch feeding stations, the secondary perch behind the primary feeding perch was not present. Jackdaw silhouettes were created by Josh Arbon, and the grass silhouette was taken from *PhyloPic* (by T. Michael Keesey).

### (b) Data collection

During the 2023 and 2024 breeding seasons (see supplementary material, figure S1 for a timeline of data collection), we deployed RFID feeding stations (*Nature Counters Ltd*, UK) at eight different locations across the two study sites (four at site Y and three at Z in 2023; three at Y and three at Z in 2024; figure 1a). Feeders were approximately 1 m above the ground and provided a seed and grain mixture ad libitum to RFID-tagged jackdaws, but not to untagged individuals. Crucially, we equipped feeding stations with an adjacent secondary RFID perch approximately 20 cm behind the primary perch (figure 1b). Here, one jackdaw could feed while another ‘queued’ behind, allowing us to quantify levels of social tolerance. The times of data loggers at feeding (primary) and queuing (secondary) perches were synchronized by a network cable to identify ‘dyadic events’, where two birds were detected at both perches simultaneously. During the study period (355 days; approx. 51 weeks in total across the 2 years), dyadic RFID feeding stations recorded visits by 722 individual jackdaws (188 females, 216 males and 318 unsexed birds; 265 of the unsexed birds were juveniles that fledged during the study across the 2 years; sex was identified via molecular sexing using blood samples). Of these 722 jackdaws, 471 individuals (121 females, 147 males and 203 unsexed) were involved in at least one dyadic event. We recorded 5512 dyadic events in total, of which 3211 events (58%) occurred in the period after the first juveniles had started using feeding stations (22 June, i.e. day approx. 174 in each year). Dyadic events involved 2433 unique dyads (1596 during the period when juveniles used feeding stations).

### (c) Relationship types

In most analyses, the key independent variable was the relationship type between two birds, classified into six categories. We considered two types of closely bonded partners: ‘pairs’ (mated partners; 46 dyads) and ‘kin’ (mostly first-order kin and a few second-order kin; 104 dyads). We classified dyads that were not pairs or kin into one of four categories assumed to represent varying levels of familiarity: ‘neighbours’ (two birds that occupied or were born in nest boxes < 50 m apart within that year and in many cases also in previous years; 102 dyads); ‘previous foraging associates’ (not neighbours, but dyads that had been observed to associate during foraging in the latter part of the previous breeding season; 353 dyads; some neighbours were also foraging associates but are classified as neighbours here); ‘site residents’ (not neighbours or foraging associates, but that were resident at the same study site; 1509 dyads); ‘other site’ (not neighbours, foraging associates or site residents, and that were resident at different study sites and encountered each other at a feeding station at one of the sites; 319 dyads). We provide further details about this classification in the supplementary material.

### (d) Statistical analyses

We processed and analysed data in *R* (v. 4.2.2) [48] via R studio (v. 2024.9.1.394) [49]. To prepare data for analyses, we used packages from the *tidyverse* (v. 2.0.0) [50] and the package *data.table* (v. 1.17.4) [51]. To test predictions, we constructed Bayesian models using the package *brms* (v. 2.22.0) [52]. For our analyses, we initially formulated a simple Directed Acyclic Graph to visualize assumptions about causal interrelationships (supplementary material, figure S2). We then constructed statistical models, defining weakly informative priors primarily for regularization. We conducted prior predictive checks to assess the suitability of priors and the model family. We fitted models with four chains and 2000 iterations each (1000 for warmup; 1000 for sampling). We assessed convergence by inspecting chain mixing via trace plots, Rhat values (< 1.01) and effective sample sizes (see supplementary material for full model summaries). When needed, we increased the default number of iterations, step size adaptation target or maximum tree depth to reduce divergent transitions and improve sampling efficiency and robustness. To evaluate model fit, we conducted posterior predictive checks. For subsequent inference and prediction using the posterior distribution, including calculation of expected values and pairwise contrasts (reported on the response scale), we used the packages *brms* and *tidybayes* (v. 3.0.7) [53]. To plot the data and posterior distributions, we used the packages *tidybayes*, *ggplot2* (v. 3.5.1) [54] and *ggpubr* (v. 0.6.0) [55]. Figures show posterior distributions of expected values, not including residual variance (grey density plots). Black dots represent the median, and black bars show the 50%, 80% and 95% credible intervals, respectively. There was no evidence of concerning collinearity among independent variables (evaluated using the package *performance* (v. 0.15.0) [56]).

To account for the fact that certain relationship types (e.g. pairs) were only possible in certain age classes, and because age classes might influence dyadic events, e.g. owing to differences in competitive ability, we added the age class combination of both birds irrespective of their order of arrival (‘adult – adult’, ‘adult – juvenile’ and ‘juvenile – juvenile’). In all models (unless mentioned otherwise), we fitted a multi-membership random effect including two individuals, as well as ‘dyad ID’ as random effects to account for individual- and dyad-level variation and dependencies in dyadic data. Multi-membership random effects have been suggested to account for interdependencies in dyadic and social network data [57]. We also fitted the position of feeding stations as a random effect to account for potential variation across locations.

#### (i) Social preference

To test the prediction that jackdaws engage in more dyadic events with bonded partners, we constructed *Model 1* (family *Poisson*). We fitted the ‘number of dyadic events per dyad’ as a dependent variable and ‘relationship type’ (six categories) as the key independent variable (truncated dependent variable with a lower bound of 1 given that we only considered dyads that participated in at least one dyadic event). As only one observation (total number of dyadic events) per dyad was used, we included the IDs of the two individuals as a multi-membership random effect, but not ‘dyad ID’. We first conducted one analysis using data from the full study period. We then repeated the analysis with a subset of the data, only including events that occurred during the time after juveniles had fledged and started using feeding stations. Most dyadic events between kin involved at least one juvenile (83%). As the period when juveniles were present was shorter, dyadic events between kin could be underestimated compared with pairs and other dyads. However, the results did not differ qualitatively, so we report the output from the analysis based on the full data collection period. To visualize how often birds engaged in dyadic events, we used the package *igraph* [58] to construct a weighted social network based on the frequency of dyadic events detected at all dyadic feeding stations (see figure 2b).

**Figure 2.**
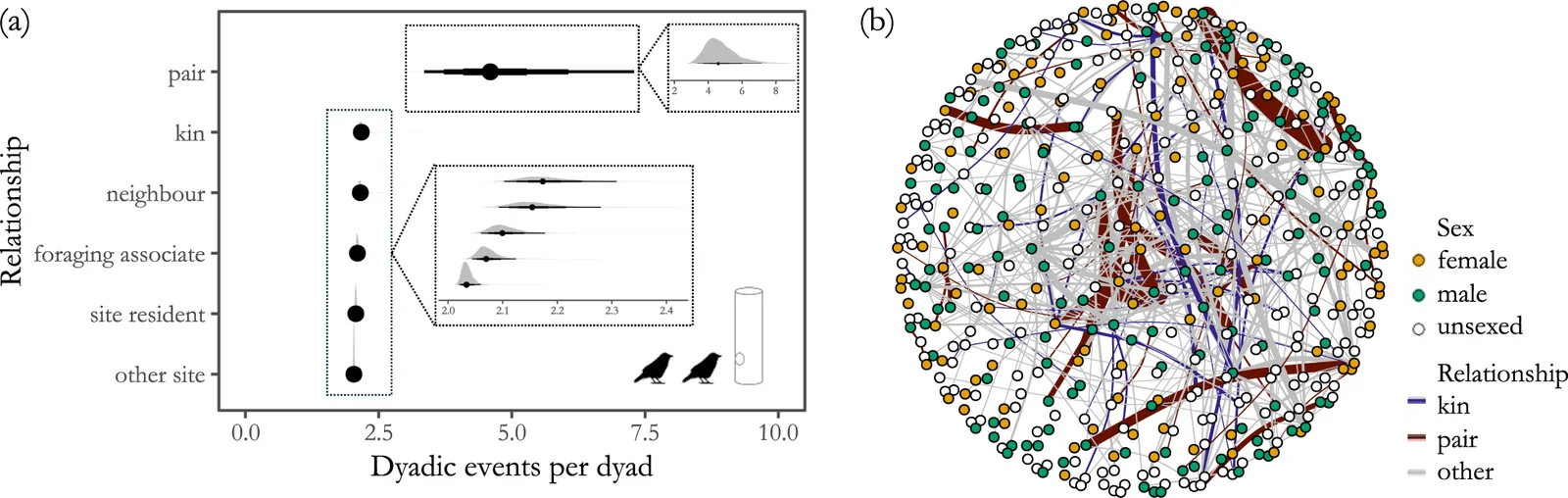
(a) Posterior distributions of the expected number of dyadic events per dyad for different relationship types across the study period. All posterior distributions are plotted on the same scale, and differences in appearance are owing to differences in posterior uncertainty between groups. Note that inserted distributions provide a ‘zoomed-in’ view, plotted on different scales to visualize differences in uncertainty between relationship types (owing to scale, distributions appear as grey lines above dots on the main figure). (b) Weighted social network of dyadic events between individuals. For visualization purposes, the network is thresholded to include edges between dyads that queued together more than three times. The thickness of edges reflects the frequency with which dyads queued together, with thicker lines indicating more dyadic events. Jackdaw silhouettes created by Josh Arbon.

#### (ii) Coordination

To test the prediction that bonded partners show greater temporal coordination, we examined the latency to arrive at feeding stations between both birds during dyadic events. We constructed *Model 2* (*Gamma* family to account for the skewed distribution of continuous data) with the ‘latency’ (the difference in the arrival time of the two birds per dyadic event (s)) as a dependent variable and the ‘relationship type’ (six categories) as an independent variable.

#### (iii) Social tolerance, benefits and costs

##### Social tolerance: duration of dyadic events

To test the prediction (a) that bonded partners perch together for longer (i.e. show greater social tolerance), we constructed *Model 3* (*Gamma* family to account for the skewed distribution of continuous data) with the duration per dyadic event (s) (time that both birds were perched together) as a dependent variable. In this variable, some very short dyadic events were classified as lasting 0 s in instances where one bird's tag was only detected once by the RFID antenna (limited by the antenna's scanning rate for tags). Given that these events were real, biologically meaningful events that had a short duration longer than 0 s, we fitted a model allowing for censored data. Observations of 0 s were thus censored and classified as being longer than 0 s and shorter than 1 s. The relationship type (six categories) was the key independent variable. As a supplementary analysis to evaluate the time available for feeding for the individual at the primary perch, we constructed another model similar to *Model 3*. Here, we used the total visit duration of the individual at the primary feeder perch during and outside of the dyadic event as a dependent variable. In this model, we accounted for the arrival latency of the individual at the secondary perch.

##### Benefits for feeding individual: probability of displacement

To test prediction (b) that individuals at primary perches would be less likely to be displaced by a third party when they were accompanied by an adult bonded partner, we constructed *Model 4* (*Bernoulli* family). The binary dependent variable of interest was whether or not individuals were displaced by a third bird, i.e. another individual that was not queueing behind them. Following Arbon *et al.* [59], we classified displacement as instances when the next individual at the perch arrived within 2 s of the focal individual leaving. This analysis only considered individuals that visited feeding stations in all three different contexts, which we fitted as the key independent variable: with an adult bonded partner, i.e. a pair-bonded mate or a parent queuing behind, with another adult queuing behind or with no bird queueing behind them. Here, we regrouped these relationship types (pair and kin as ‘bonded’, and all other relationship types as ‘other’) to counterbalance the much larger sample size that was available for solo events. In the two different contexts of dyadic events, we only included those in which the queueing individual was an adult, assuming that juveniles had only a minor impact on the probability that the feeding individual would be displaced by a third party. We fitted the age class combination of the current and the next visitor as an additional independent variable (conserving information about the order of arrival). We only included instances where the next visitor (i.e. the potential displacer) was a different individual than the current visitor (i.e. the potentially displaced individual). For solo events, we only considered cases where the second bird arrived within 300 s (5 minutes) of the feeding bird leaving. This was because displacement could actually occur if other birds were nearby. In this model, the multi-membership and dyad ID random effects included the bird at the primary feeder perch and the next bird that would perch at the primary feeder perch thereafter.

##### Consequences for queueing individual

Finally, we examined the probability that the queuing individual would be the next individual at the primary perch after the dyadic event had ended (prediction (c)). In principle, individuals might allow their bonded partner to queue behind them to feed after them, but individuals queuing behind social partners may also accept paying the time cost of foregoing feeding. To test this, we constructed *Model 5* (*Bernoulli* family). As a dependent variable, for each dyadic event, we identified whether or not the queuing individual was the next individual at the primary perch after the individual at the primary perch had left. As a main independent variable, we fitted the relationship type of the two birds (six categories). In addition to the random effects mentioned above, here we added the individual ID of the jackdaw that was perched at the primary perch after the dyadic event had ended.

#### (iv) Social learning

Across the 2 years of this study, 265 juveniles were detected at dyadic RFID feeding stations. Of these, 180 visited at least one primary perch of a feeding station, so could feed directly (remaining juveniles were only detected at secondary perches). Juveniles could visit multiple feeding stations, and we defined the unit of analysis as a juvenile's first visit to the primary perch of a given focal feeding station. This resulted in 255 first visits across 180 juveniles, with some juveniles contributing more than one data point. For the analyses, we only considered juveniles that had been ringed before the first juvenile visited a feeding station (*N* = 127 juveniles). This was to ensure that all first visits were detected reliably because juveniles that were ringed later could have been visiting feeding stations before being tagged.

We inferred the ‘following’ events between parents and offspring that could serve as social learning opportunities from the sequence of visits to feeding stations. We defined the following event as an instance in which a juvenile made its first visit to a given focal feeding station and was detected after one of its parents, with no other bird visiting in between. This definition of the following events based on the sequence of visits was consistent with the latency of arrival at feeding stations: during the following events with a parent, juveniles arrived shortly after their parent had left (median = 5.76 s; in 91% of the cases the latency was less than 90 s). By contrast, the latency of arrival was much longer when juveniles visited for the first time after another bird had left (median = 203.14 s). Of 191 instances in which juveniles (*N* = 127) visited a given feeding station for the first time, they directly followed their parents in 55 instances (29%). At the individual level, 47 of 127 (37%) juveniles followed their parents at least once during a first visit to a focal feeding station. The other 80 individuals were never observed directly following parents (for 31 juveniles, parents had not been detected at feeding stations).

To test whether juveniles might use opportunities to learn socially about locations and the use of feeding stations by following their parents, we constructed two models. For each first visit by a juvenile at a given focal feeding station, we assessed whether or not they had followed their parents directly. In both models, we fitted an independent variable that included three ‘categories’, indicating the ‘following status’ of a juvenile on their first visit at a focal feeding station: juveniles that visited the focal feeding station directly after their parents (‘followed’); the same juveniles that visited at least one other feeding station, but not the ‘focal’ one, after their parents (‘sometimes independent’); juveniles that never visited a feeding station right after their parents (‘always independent’). The distinction between the first two groups was to test whether juveniles who sometimes followed their parents showed different behaviour in cases where their parents were not immediately present.

In the first model, we only considered each juvenile's first visit to a given focal feeding station. If parents are an important source of social information, we predicted that juveniles following their parents directly would visit focal feeding stations earlier than juveniles who made their first visit independently of a parent (*Model 6*, *Gamma* family). The dependent variable of interest was the day in the year that juveniles first visited a given focal feeding station (the first juvenile was detected at feeding stations on 22 June in both years, i.e. day 174). In the second model, we considered how first visits would predict subsequent visit frequency at feeding stations. Specifically, we tested whether juveniles that ‘followed’ (i.e. whose first visit followed immediately after a parent) went on to visit the focal feeding station more frequently compared with juveniles that made their first visit independently (*Model 7*, *Poisson* family; truncated dependent variable with a lower bound of 1 given that we only considered focal feeding stations that a given juvenile visited at least once). In both models, we fitted the day that a given brood had been ringed as a covariate (*z*-transformed). This was to account for variation in the timing of fledging, which determined when juveniles could possibly visit feeding stations. We added the individual ID of juveniles and the location of feeding stations as random effects.

## 3. Results

### (a) Social preference

Jackdaws engaged in more dyadic events with bonded partners (particularly pair-bonded partners) than with others (*Model 1*; figure 2; supplementary material, tables S1 and S2). Pairs participated in more dyadic events than other relationship types (posterior expected number of dyadic events per dyad = 4.60, 95% credible interval (CrI) [3.36, 7.56]), followed by kin (2.17, 95% CrI [2.10, 2.32]), neighbours (2.15, 95% CrI [2.09, 2.28]), foraging associates (2.10, 95% CrI [2.06, 2.18]), site residents (2.07, 95% CrI [2.04, 2.12]) and birds from different sites (2.03, 95% CrI [2.02, 2.06]). Pair-bonded mates engaged in more dyadic events than all other relationship types, as suggested by pairwise contrasts (100% of the posterior for all pairwise comparisons between pairs and other dyads; supplementary material, table S2). Kin also participated in more dyadic events than all other relationship types (except pairs), but the difference was much smaller than between pairs and other dyads (figure 2a; supplementary material, table S2).

### (b) Coordination

Bonded partners (pairs and kin) were more coordinated during foraging (figure 3; supplementary material, tables S3 and S4). The estimated arrival latency was lowest for pairs (posterior expected latency = 17.87 s, 95% CrI [14.50, 22.01]) and kin (18.11, 95% CrI [14.78, 22.51]), followed by dyads from different study sites (22.73, 95% CrI [18.60, 28.05]), neighbours (25.40, 95% CrI [20.33, 32.12]), site residents (25.45, 95% CrI [21.35, 30.68]) and foraging associates (26.70, 95% CrI [22.20, 32.55]). Arrival latency was estimated to be nearly 25% shorter for pairs and kin compared with the other relationship types (posterior probability > 99%; supplementary material, table S4). By contrast, arrival latencies between kin and pair dyads did not differ clearly (posterior probability 57% that arrival latency was shorter for pairs).

**Figure 3.**
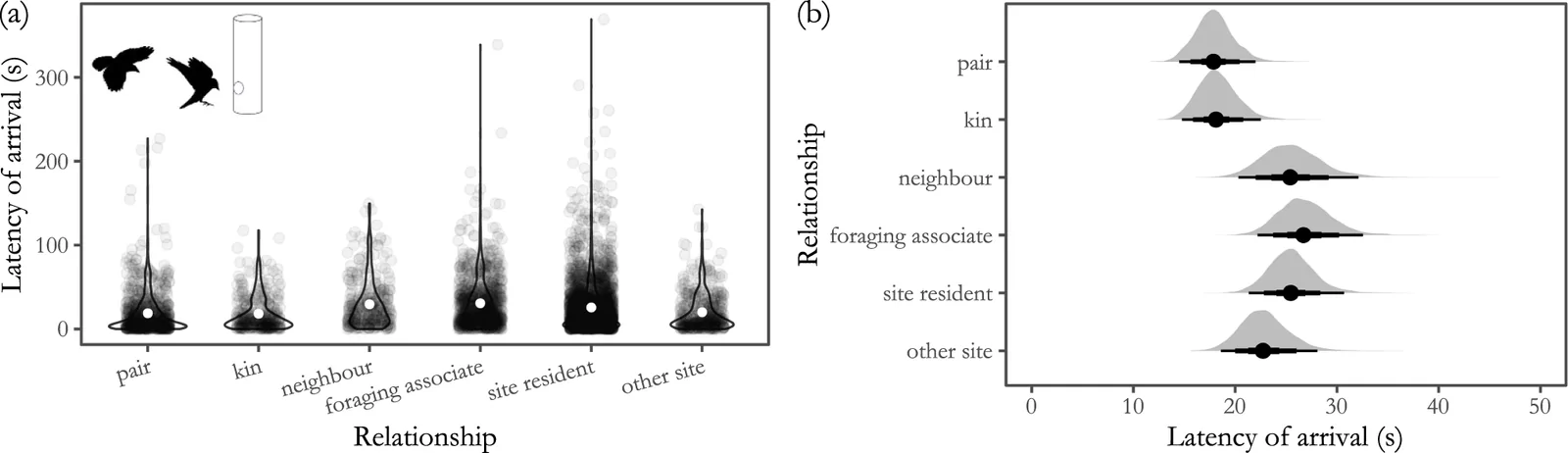
(a) Observed latency of arrival (s) between two individuals engaging in dyadic events (each data point shows one dyadic event, and white circles show the mean latency per relationship type). (b) Posterior distributions of the expected latency of arrival (s) per relationship type. Jackdaw silhouettes created by Josh Arbon.

### (c) Social tolerance, benefits and costs

#### (i) Social tolerance: duration of dyadic events

Dyadic events lasted longer between bonded partners than between other dyads (figure 4a,b; supplementary material, table S5 and S6). Dyadic events were at least twice as long for pairs (posterior expected dyadic event duration = 10.23 s, 95% CrI [7.70, 13.96]) and for kin (9.35, 95% CrI [6.94, 12.63]), compared with site residents (4.63, 95% CrI [3.73, 5.88]), dyads from different sites (4.30, 95% CrI [3.35, 5.67]), neighbours (3.53, 95% CrI [2.64, 4.77]) and foraging associates (2.98, 95% CrI [2.35, 3.88]). Dyadic events were estimated to last longer for pairs and for kin than for all other relationship types, as indicated by pairwise contrasts (100% of the posterior for each of the comparisons; supplementary material, table S6). Additionally, dyadic events tended to be slightly longer for pairs than for kin, but this difference was more uncertain (75% posterior probability). Dyadic events were also longer between site residents and dyads from different sites than they were for neighbours and foraging associates (supplementary material, table S6). There was no clear difference in dyadic event duration between site residents and dyads from different sites, and also no difference in duration between neighbours and foraging associates (supplementary material, table S6). The results were qualitatively similar when repeating the analysis using the total visit duration of the individual at the primary feeding station, accounting for the arrival latency between both birds (supplementary material, table S7). Video validation showed that visit durations at dyadic feeding stations were strongly correlated with the number of feeding bouts, suggesting that accessing feeding stations for longer was likely to result in higher food intake (supplementary material, figure S3).

**Figure 4.**
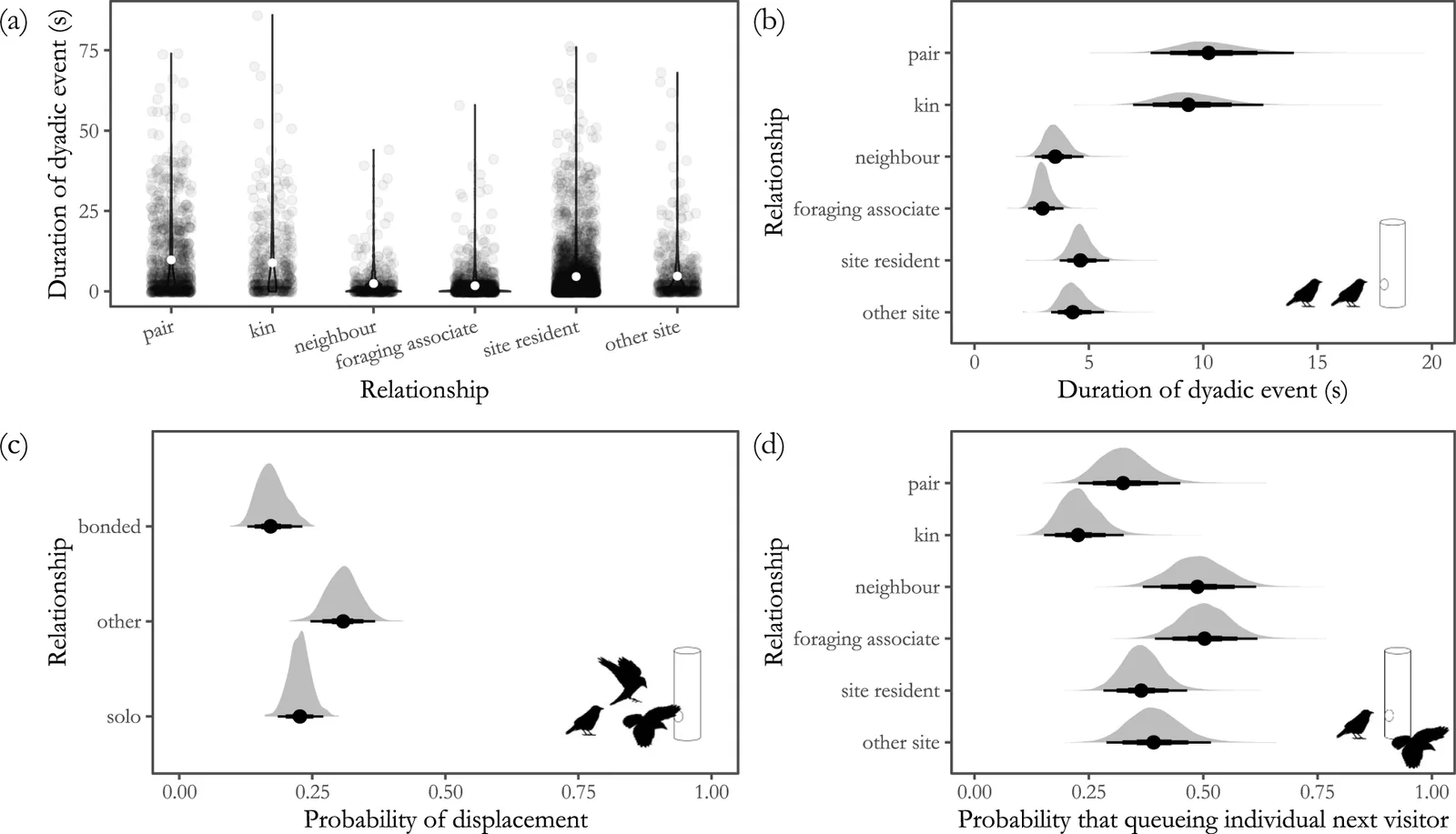
(a) Observed duration of dyadic events (s) for different relationship types. Each data point reflects one dyadic event between two individuals. White circles denote mean durations per relationship type. (b) Posterior distributions of the expected dyadic event duration (s) per relationship type. (c) Posterior distributions of the expected probability of being displaced across three different contexts (with an adult bonded partner queueing behind, with another adult queuing behind, with no other bird queuing behind). (d) Posterior distributions of the expected probability that the queueing bird would be the next individual at the primary feeder perch after the dyadic event had ended. Jackdaw silhouettes created and sketches inspired by Josh Arbon.

#### (ii) Benefits for feeding individual: probability of displacement

The social context in which individuals visited primary perches of dyadic feeding stations was associated with the probability of being displaced by a third party in the case of dyadic events or by the next visiting bird within 5 min in the case of solo visits (figure 4c; supplementary material, table S8). The estimated probability of being displaced was lowest when individuals were accompanied by an adult bonded partner (pair-bonded mate or parent) queueing behind them (posterior expected probability = 0.17, 95% CrI [0.13, 0.23]), intermediate when no other bird was queueing behind (0.23, 95% CrI [0.18, 0.27]) and highest when another adult was queuing behind them (0.31, 95% CrI [0.25, 0.37]). Pairwise contrasts further supported this finding: individuals were less likely to be displaced by a third party when a bonded partner was queuing behind them compared with when another adult (*bonded versus other*, expected difference in probability = –0.13, 95% CrI [–0.19, –0.08]; 100% of the posterior) or when no other bird was behind them (*bonded versus solo*, –0.05, 95% CrI [–0.09, –0.01]; 99% of the posterior). Individuals were also less likely to be displaced when no other bird was present compared with when another (non-bonded) adult was queuing behind them (*other versus solo*, 0.08, 95% CrI [0.04, 0.12]; 100% of the posterior).

#### (iii) Consequences for queuing individual

The queueing individual was detected at the primary perch after the dyadic event had ended in less than 50% of dyadic events (figure 4d; supplementary material, tables S9 and S10). The estimated probability that the queuing individual would be the next bird to access the primary perch was lowest for dyadic events between kin (posterior expected probability = 0.23, 95% CrI [0.15, 0.33]), followed by pairs (0.32, 95% CrI [0.23, 0.45]). The probability that a queueing individual would go on to occupy the primary perch after the dyadic event was similar between site residents (0.36, 95% CrI [0.28, 0.46]) and dyads from different sites (0.39, 95% CrI [0.29, 0.52]). Finally, individuals queueing behind neighbours (0.49, 95% CrI [0.37, 0.62]) or foraging associates (0.50, 95% CrI [0.39, 0.62]) had the highest probability of accessing the primary perch after the dyadic event had ended (see supplementary material, table S10 for all pairwise contrasts).

### (d) Social learning

Juveniles made their first visit at focal feeding stations earlier, when they followed their parents directly (posterior expected day of the year = 185.36, 95% CrI [180.42, 191.95]) compared with feeding stations where they did not follow their parents (195.91, 95% CrI [189.00, 203.35]; *sometimes independent – followed*, expected difference = 10.30 days, 95% CrI [3.92, 17.12]; 100% of the posterior; figure 5a,b; supplementary material, table S11). Juveniles who followed their parents also visited focal feeding stations earlier than other juveniles who never followed their parents (196.83, 95% CrI [191.76, 202.71]; *always independent –* *followed*, 11.34, 95% CrI [8.23, 14.43]; 100% of the posterior). However, when juveniles who followed their parents at some feeding stations visited other focal feeding stations independently, they did not visit them earlier than those juveniles who never followed their parents (*always independent – sometimes independent*, 0.89, 95% CrI [–5.40, 7.20]; 62% of the posterior). Similarly, juveniles visited given focal feeding stations more frequently later on if they had followed their parents on their first visit (posterior expected number of visits = 4.98, 95% CrI [2.58, 15.29]) compared with those feeding stations where they had not followed their parents (3.39, 95% CrI [2.31, 8.92]; *sometimes independent – followed*, expected difference in number of visits = –1.56, 95% CrI [–6.51, –0.26]; 100% of the posterior; figure 5c,d; supplementary material, table S12). Juveniles who had followed their parents to focal feeding stations also visited those feeding stations more frequently than juveniles who had never followed their parents (3.06, 95% CrI [2.25, 7.06]; *always independent – followed*, –1.82, 95% CrI [–8.96, –0.22]; 100% of the posterior). Juveniles who had followed their parents at some feeding stations tended to visit those feeding stations, where they had not followed more frequently than juveniles who had never followed their parents, but this difference was uncertain (*always independent – sometimes independent*, –0.29, 95% CrI [–2.94, 0.71]; 78% of the posterior).

**Figure 5.**
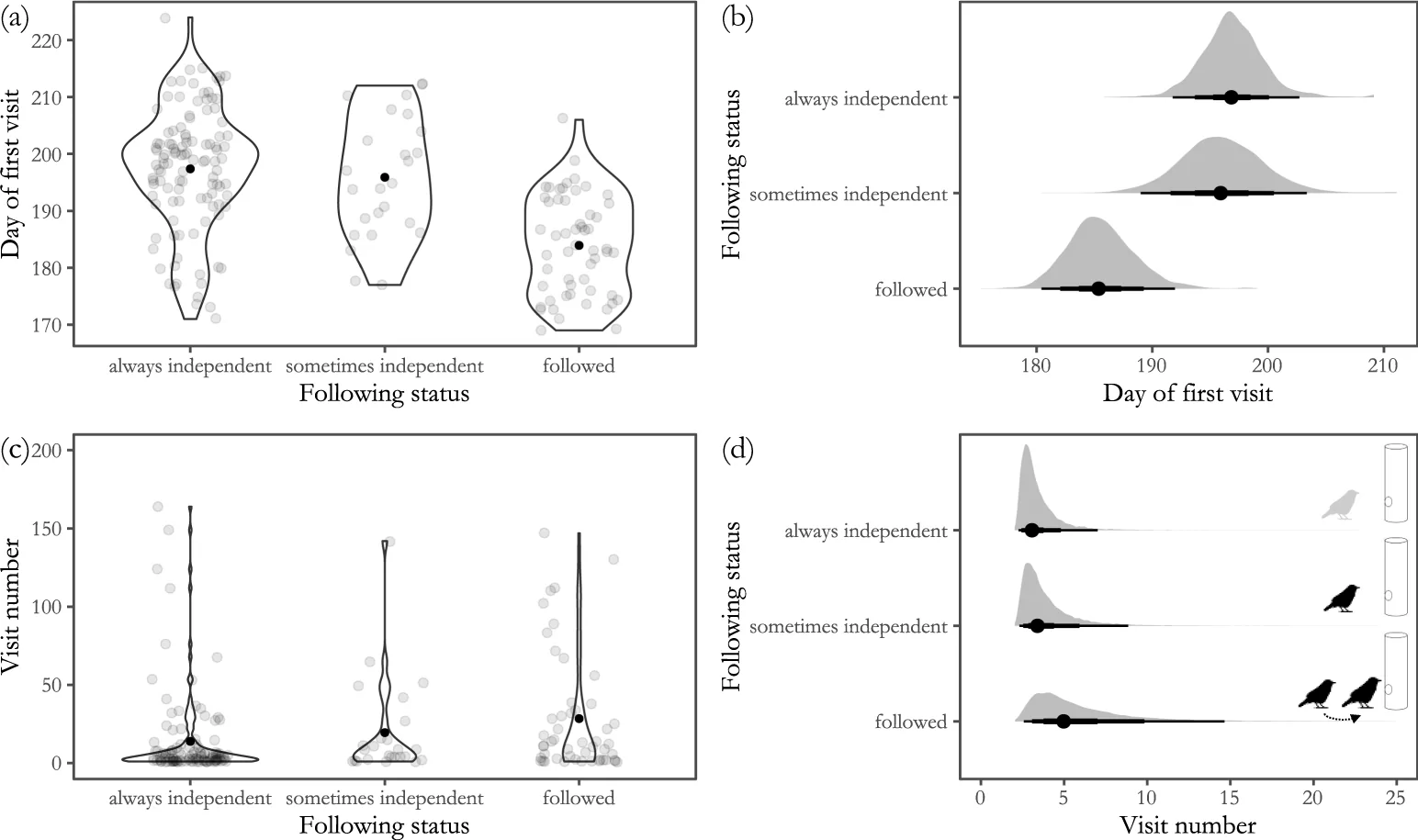
Use of feeding stations by juveniles depends on whether they follow their parents on their first visit. (a) and (b) The day on which juveniles made their first visit to a given feeding station. (c) and (d) Number of visits made by juveniles at feeding stations. Panel (a) and (c) show the raw data, whereby each data point corresponds to an instance of a juvenile visiting a focal feeding station, i.e. when juveniles visited multiple feeding stations, they contributed a data point for each feeding station they visited. The ‘following status’ represents three different scenarios: juveniles never followed their parents (‘independent (always)’; grey jackdaw silhouette in (d)), juveniles sometimes followed their parents to feeding stations but visited the focal feeding station independently (‘independent (some)’; black silhouette of one jackdaw in (d)) and the same juveniles followed their parents at a focal feeding station (‘followed’; black silhouettes of two jackdaws and arrow in (d)). Panel (b) and (d) show the posterior distribution of the expected value per scenario. Jackdaw silhouettes created by Josh Arbon.

## 4. Discussion

We tested the overarching hypothesis that jackdaws' social relationships shape their decisions and encounters at monopolizable feeding stations, with consequences for acquiring food and information. As predicted, dyadic events involving bonded partners were more frequent, more coordinated and longer. Thus, compared with other dyads, bonded partners potentially benefitted from greater food intake through reduced probability of displacement (for the bird at the primary perch), and from social learning opportunities (for juveniles), but queueing individuals also incurred short-term time costs.

Bonded partners visited feeding stations together more frequently, arrived with shorter latencies and queued in proximity for longer compared with other dyads. Being regularly accompanied by and coordinating foraging activities with bonded partners can potentially make foraging outcomes at monopolizable resources more predictable, for example, in terms of accessing and defending resources. This is particularly relevant in social systems characterized by dynamic, potentially unpredictable and competitive social interactions, as observed in jackdaws [60]. By coordinating foraging activities with high social tolerance, individuals may be reliably accompanied by a partner that could provide social support when faced with competition. Indeed, our findings suggest that social partners may not merely tolerate each other more but benefit directly from each other's presence, reducing the probability of displacement and enhancing access to contested food resources. Therefore, jackdaws likely benefitted from their decision to forage alongside bonded partners.

Our findings are consistent with reports from other corvids. In rooks *(Corvus frugilegus)*, high social tolerance within mated pairs facilitated scrounging for food [61] and in Northwestern crows *(Corvus caurinus)*, individuals were less aggressive towards close relatives [62]. In non-breeder groups of common ravens, bonded individuals competed for food more successfully even when their partner was not immediately present [63]. In our study, bonded partners obtained immediate foraging-related benefits through social tolerance and potentially social support when queuing, resulting in a decreased probability of being displaced. Such short-term foraging benefits may lead to longer-term fitness benefits, such as better body condition and health, and potentially greater survival and/or reproductive success (e.g. [64]). This could be particularly relevant for juveniles who lack competitive ability and need to ensure consistent food intake over the course of their development. We can also expect that greater social tolerance among kin and mated partners provides indirect fitness benefits, owing to kin selection [65] and high fitness interdependence between (highly genetically) monogamous mated partners [34,66], respectively.

We also found that jackdaws incurred short-term time costs, foregoing foraging to queue behind and remain with bonded partners. Individuals who queued behind bonded partners during dyadic events were less likely to access the primary feeder perch after the dyadic event had ended compared with those who had queued behind other, non-bonded individuals. Therefore, the time cost incurred by bonded queueing individuals often extends beyond the duration of the dyadic event. Reflecting the potential benefits discussed above, bonded partners may be willing to incur such time costs, owing to high fitness interdependence and inclusive fitness. Future work could investigate the temporal dynamics of queuing in our system; for example, if and how tolerance changes with variable foraging payoffs [59,67,68] and whether individuals might alternate roles, taking turns to feed and queue in a form of reciprocal cooperation. Such turn-taking could potentially help stabilize cooperative partnerships [69], reinforcing the mutual benefits of maintaining close social bonds.

Beyond closely bonded social partners (pairs and kin), the frequency of social foraging events and their associated benefits and costs may be linked to the degree of familiarity between individuals. Dyads with relatively higher familiarity (neighbours, foraging associates) engaged in slightly more dyadic events than less familiar dyads (site residents and birds from different sites). They did not, however, show higher coordination or social tolerance, and the duration of dyadic events between neighbours and foraging associates was even shorter than that between less familiar dyads. These results are somewhat unexpected, given that animals commonly show greater social tolerance towards more familiar individuals. For example, animals often avoid the costs of continued competition and aggression by showing increased tolerance towards familiar neighbours (‘dear enemy effect’; e.g. [70]). One possibility is that familiar individuals resolve queueing interactions more rapidly because prior interactions make outcomes more predictable. Consistent with this idea, the probability that a queueing bird subsequently accessed the primary feeder perch was highest when familiar neighbours or foraging associates queued together. This raises the possibility that increased access to the primary feeder perch compensates for the shorter duration of dyadic events between familiar individuals. Understanding how the cumulative costs and benefits of such social decisions emerge over time is a clear priority for future work.

Our findings also suggest that, as predicted, close social associations and tolerance between parents and offspring may have promoted social learning about feeding stations by juveniles. Because we studied juveniles shortly after fledging, they were initially naïve about the distribution of food resources. We found that juveniles that first visited a feeding station directly after a parent did so earlier and subsequently revisited that feeder more often than if the first visit occurred independently of a parent. This indicates that juveniles who were recorded following parents did not simply have a greater general tendency to visit feeding stations earlier and more often. Rather, these effects occurred specifically when a parent had visited a feeding station directly beforehand, thus providing an opportunity for social information transmission. By contrast, juveniles that sometimes followed their parents did not visit other feeding stations independently earlier than juveniles who never followed their parents and only tended to visit them slightly more often, although this difference was uncertain. Whether juveniles generalize information learned from parents at one location to others, therefore, remains inconclusive and requires further investigation. Nevertheless, regardless of whether such generalization occurs, our results suggest that close associations with parents facilitate learning about feeder locations and access. Such social learning early in life may have long-lasting consequences. For example, in blue tits *(Cyanistes caeruleus)* and great tits, foraging behaviour learned from foster parents persisted throughout life [71]. In long-lived species with extended parental care, such as corvids, vertical cultural transmission of social information may be particularly valuable [72]. Obtaining information by associating with more knowledgeable individuals could also be one of the underlying causal mechanisms linking sociality to long-term fitness.

Overall, we showed that close long-term social relationships shape the likelihood and outcomes of fine-scale dyadic foraging encounters in a dynamic social system of wild jackdaws. In this social system, characterized by high levels of fission-fusion dynamics [42], individuals may be faced with social uncertainty and competition owing to the variable and transient nature of social interactions with a high number of different other individuals. Coordinating with and tolerating bonded social partners with shared interests can enable individuals to manage uncertainty and increase short-term access to food and information. Thus, through their daily social information use and decision-making, individuals may construct their own immediate social niches [73]. Through their decisions, individuals may balance the benefits and costs of sociality and maximize short-term payoffs, with important effects on long-term fitness.

## Supplementary Material



## Data Availability

Data and code required to reproduce the analyses are available on Zenodo [75]. Supplementary material is available online [76].
