# Supplementary material for: Social relationships promote access to food and information in wild jackdaws
Source: Proc Biol Sci. 2026 Oct 7;293(2080):20261462. doi: 10.1098/rspb.2026.1462 (PMC13641922; doi:10.1098/rspb.2026.1462)
Supplement: Supplementary file 1 [file rspb.2026.1462.zip › rspb.2026.1462/rspb20261462_si_001.pdf]

Social relationships promote access to food and information in wild jackdaws

Luca G. Hahn[1,2]*, Guillam E. McIvor[1,3], Ines Fürtbauer[2], Andrew J. King[2], Alex Thornton[1]*
1 Centre for Ecology and Conservation, University of Exeter, Penryn, United Kingdom
2 Department of Biosciences, Swansea University, Swansea, United Kingdom
3 Department of Behavioral and Cognitive Biology, University of Vienna, Vienna, Austria
**Corresponding authors: luca.hahn8@gmail.com, alex.thornton@exeter.ac.uk*

Proceedings of the Royal Society B: Biological Sciences

10.1098/rspb. 2026-1462

Supplementary Material

Methods

Data collection and processing

We conducted this study as part of the *Cornish Jackdaw Project*, a long-term project that collects detailed information about the life history and behaviour of thousands of individually recognisable jackdaws. Jackdaws are opportunistic generalists that can exploit a range of food sources, from invertebrates to anthropogenic food. In our study populations, there is substantial inter- and intra-individual variation in the propensity to use supplemental feeding stations (1) but overall supplementary food likely only contributes a minor proportion of jackdaws' total food intake. Given the mild, moist Cornish climate, food is not scarce during the spring and summer, but jackdaws may potentially benefit from supplemental food, especially during the likely energetically demanding breeding period.

We equipped feeding stations with a data logger (including an SD card), an *RFID* antenna within a perch, and an *RFID*-governed opening mechanism restricted and executed by a servo. We programmed loggers such that a servo would open a small door at the front of the feeding station for jackdaws fitted with a functional *RFID*-tag. Throughout the study period, we checked feeders for functionality daily, and we performed data transfer and battery changes every three days or whenever required. *RFID* antennae can scan for passive tags approximately once per second, meaning that if a bird sits on the perch, multiple reads can be recorded.

RFID loggers at feeding stations generated text files (*.txt* format) that were saved onto SD cards and contained a temporal data stream including the date, time of day, and individual identity of each visiting jackdaw (individual tags were represented in a 10-character hexadecimal format). We then concatenated files to create one overall data frame for further data processing and analyses using packages from the *tidyverse* (2), including the packages *dplyr* (3)*, lubridate* (4)*,* and *stringi* (5). In this study, multiple *RFID* reads

of the same unique tag within 10 seconds of each other (as an arbitrary but reasonable threshold) were classified as one 'visit'. As soon as there was a period of 10 seconds without a read from a particular individual, the last read was therefore interpreted as the end of one visit. We cleaned the raw data where necessary, such as in instances of rare time slips by data loggers. For instance, in a few cases, the year had to be corrected. Considering the two different study sites, 496 and 438 individuals visited feeding stations at site *Y*, and site *Z* respectively (216 individuals visited feeding stations at both site *Y* and site *Z*). Throughout the study period, dyadic *RFID* feeding stations recorded 174,824 'visits' from 722 jackdaws fitted with *RFID* tags, of which 124,208 were at primary perches (i.e., the main feeder perch, including feeding stations without a secondary perch), and 50,616 were at secondary perches (i.e., the perch fitted behind the feeders). The proportion of jackdaws in the population that are fitted with colour rings is relatively high. For example, in 2023, (the first year of data collection for this study), we captured 194 adults, of which 139 (72%) were already marked. All juveniles born in the project's nestboxes and in other accessible, nearby cavities (e.g. tree holes etc.) are ringed approximately one week before fledging.

We then used this concatenated data frame containing all visits at *RFID* feeding stations to identify dyadic events based on the exact timestamps of visits, using packages from the *tidyverse* and the package *data.table* (6). For a dyadic event to occur, two birds had to be perched at the same feeding station simultaneously, with one bird occupying the primary feeder perch and the other bird occupying the secondary queueing perch. In some instances, a bird occupying the primary feeder perch could be involved in more than one dyadic event if during its visit at least two different birds occupied the secondary perch consecutively. We refer to the bird arriving at one of the two perches first as the "initiator", and the second bird as the "joiner". In approximately 80% of dyadic events, the initiator sat on the primary feeder perch and the joiner on the secondary perch; for the other 20% of events the positions were reversed.

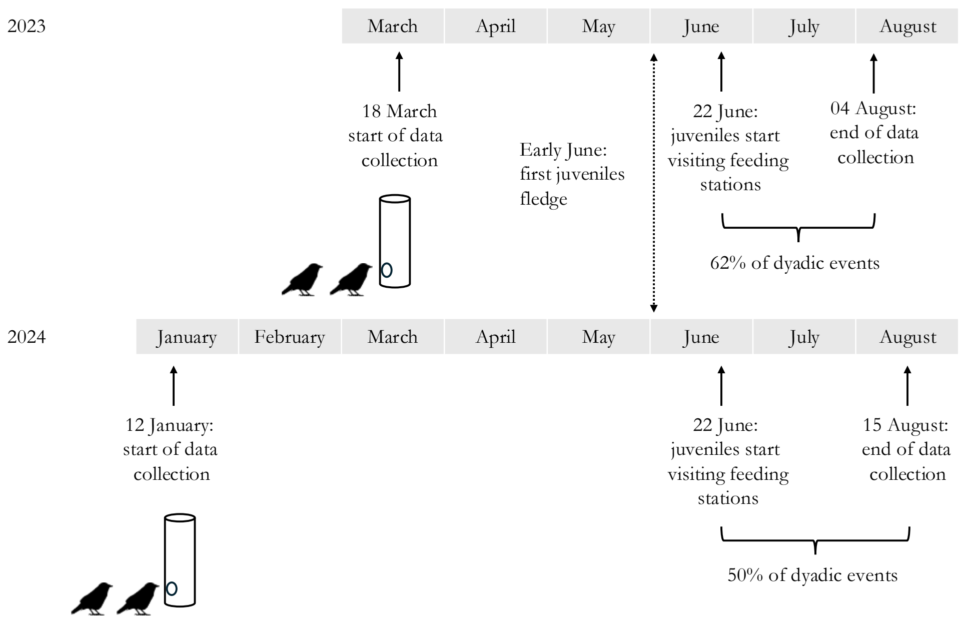

Figure S1 | Timeline of the data collection period across two years. The first few months of the data collection period (until early May) primarily served to habituate jackdaws to the presence of feeding stations (feeding stations of this type had been used during breeding seasons since 2021). Jackdaws at our study sites tend to start using supplemental feeding stations more routinely in early May, with visit rates declining again towards late July. Juveniles enter the population within the first few weeks of June and are ringed approximately one week before fledging. In both years, the first juvenile using feeding stations was detected on the 22nd of June.

Relationship types

- We identified pairs and kin primarily by monitoring nest boxes and other nearby cavities (jackdaws are cavity nesters that readily accept nest boxes for breeding). The vast majority of dyads (95% of kin dyads) and dyadic events involving kin were between first-order kin, i.e., parents and offspring (nearly 80% of events between first-order kin) or between siblings. On rare occasions (less than 1% of all dyadic events), second-order kin, such as grandparents and grand-offspring, encountered each other. Two of the 46 identified pairs had been formed between kin, but in this study, we identified them as pairs and not kin. We identified kin relationships via a social, not a genetic pedigree.

- Similarly, we identified breeding neighbours by monitoring nest-box occupancy. We assumed neighbouring pairs breeding within approximately 50 m of each other in the years of data collection to be familiar with each other. Jackdaws are highly faithful to their nest cavity and often nest in the same cavity over multiple years, so in many instances neighbours had likely known each other already for an extended period of several years. In cases where neighbours were nesting next to each other for the first time, they still had the opportunity to become more familiar with each other

through spatial proximity over several months. We assumed this to be the case given that jackdaws often tend to secure and defend their nest cavity over several months before breeding.

- For the fourth relationship category, we considered dyads that were not closely bonded partners (kin and pairs) but that had already associated during foraging at the end of the breeding season (July) in the year before. We identified foraging associations by constructing a social network from data collected at four feeding stations (two at each site) in July 2022 and July 2023. In 2022, feeding stations were not fitted with a secondary perch yet, and we therefore only used data from the primary perches to construct a comparable network in 2023. We employed the gambit of the group approach (7), using a *Gaussian Mixture Model* (8) via the package *asnipe* (version 1.1.17) (9). From the resulting group-by-individual matrix we obtained a weighted adjacency matrix with associations calculated via the simple ratio index (10). We then defined foraging associates in a given year as dyads that had an edge weight larger than 0 in the year before (i.e., that were associated at least once). We classified dyads with a previous foraging association that also occupied nest boxes within 50 m as neighbours. In this study, we considered neighbours and foraging associates as 'intermediate' relationships between closely bonded partners and less familiar individuals.

Finally, we classified the last two relationship categories based on general site residency, using information from *RFID* feeder data. Many dyads that were not closely bonded (pairs, kin) and that were also not breeding neighbours or foraging associates were resident at the same study sites and were more likely to have encountered each other before. By contrast, some individuals also moved between the study sites, which resulted in encounters between two birds that were likely less familiar with each other.

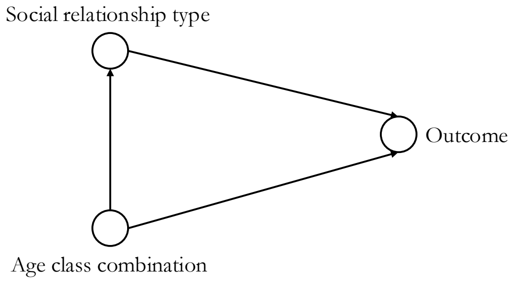

Figure S2 | Simple *Directed Acyclic Graph (DAG)* illustrating the main assumptions about variables in our models. The relationship type between two birds (six categories) was the key independent variable of interest. We tested the association between the relationship type of two birds and different outcomes, assuming a causal influence of the former on the latter. In our models, we accounted for the age class combination of both birds, assuming it might be a confounding factor that could influence both the relationship type (certain relationship types are only possible within certain age classes, such as pairs), and the outcomes.

Visit duration and food intake

To validate that visit duration obtained from *RFID* data corresponded to food intake (and as part of another experiment), we recorded video footage for 221 visits at *RFID* feeding stations using camcorders *(Panasonic)* at feeders. For each visit of a jackdaw at the *RFID* feeding station, we recorded the duration of the visit and the number of distinct feeding bouts during that visit (separate head movements towards the feeder). We examined this relationship between the number of feeding bouts (dependent variable) and visit duration (s) (independent variable) using a GLM with Poisson distribution.

Results

Visit duration and food intake

Longer visit duration at *RFID* feeding stations corresponded to more feeding bouts, i.e. greater food intake (β = 0.016, *SE* = 0.001, χ^2 = 840.9, *df* = 1, *P* < 0.001; Figure S3).

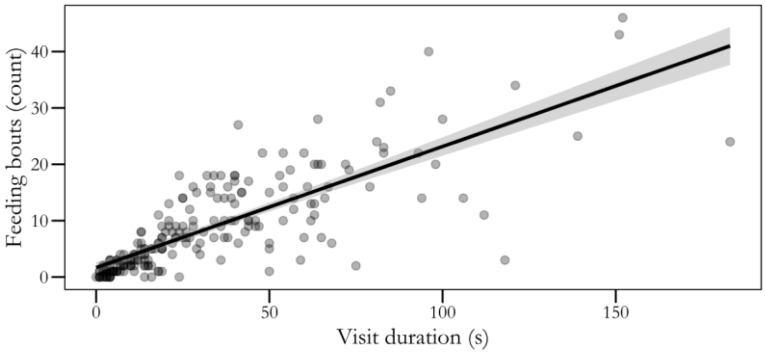

Figure S3 | Number of feeding bouts during visits at feeding stations as a function of visit duration (s).

Supplementary information from analyses reported in main manuscript

Table S1 | Summary of *Model 1*: social preference (number of dyadic events)

Coefficient	*Est.*	*Est. Error*	*Lower 95% CrI*	*Upper 95% CrI*	*Rhat*	*Bulk ESS*	*Tail ESS*
Intercept	-1.24	0.26	-1.74	-0.71	1.00	1200	1724
relationship – kin	0.52	0.09	0.34	0.70	1.00	3170	3027
relationship – neighbour	0.42	0.08	0.26	0.58	1.00	3242	3386
relationship – other site	-1.08	0.10	-1.29	-0.89	1.00	3419	2907
relationship – pair	2.72	0.07	2.59	2.85	1.00	3337	3229
relationship – site resident	-0.36	0.06	-0.48	-0.23	1.00	2464	2827
age class combination – adult and juvenile	-0.11	0.10	-0.29	0.08	1.00	1494	2045
age class combination – juvenile and juvenile	0.45	0.19	0.08	0.82	1.00	1409	1590
multi-membership (random effect)	1.70	0.10	1.51	1.90	1.01	649	1110
position (random effect)	0.59	0.22	0.29	1.11	1.01	1365	2011

Table S2 | Pairwise contrasts for *Model 1*: social preference (number of dual events)

Pairwise contrast	*Median*	*Mean*	*Lower 95% CrI*	*Upper 95% CrI*	*Posterior Probability*
foraging associate - other site	0.07	0.07	0.04	0.12	100%
foraging associate - site resident	0.03	0.03	0.01	0.06	100%
kin - foraging associate	0.07	0.08	0.04	0.15	100%
kin - neighbour	0.02	0.02	-0.02	0.07	84.9%
kin - other site	0.14	0.15	0.08	0.26	100%
kin - site resident	0.10	0.11	0.06	0.20	100%
neighbour - foraging associate	0.05	0.06	0.03	0.11	100%
neighbour - other site	0.12	0.13	0.07	0.23	100%
neighbour - site resident	0.08	0.09	0.05	0.16	100%
pair - foraging associate	2.50	2.71	1.29	5.37	100%
pair - kin	2.43	2.64	1.24	5.19	100%
pair - neighbour	2.45	2.66	1.26	5.28	100%
pair - other site	2.57	2.78	1.33	5.49	100%
pair - site resident	2.53	2.75	1.31	5.42	100%
site resident - other site	0.04	0.04	0.02	0.07	100%

Table S3 | Summary of *Model 2*: coordination (latency of arrival)

Coefficient	*Est.*	*Est. Error*	*Lower 95% CrI*	*Upper 95% CrI*	*Rhat*	*Bulk ESS*	*Tail ESS*
Intercept	3.29	0.10	3.10	3.48	1.00	1199	1755
relationship – kin	-0.38	0.09	-0.55	-0.22	1.00	2244	3189
relationship – neighbour	-0.05	0.08	-0.21	0.11	1.00	2132	2520
relationship – other site	-0.16	0.07	-0.31	-0.02	1.00	2079	2824
relationship – pair	-0.40	0.07	-0.55	-0.27	1.00	2593	2886
relationship – site resident	-0.05	0.05	-0.15	0.05	1.00	1469	2317
age class combination – adult and juvenile	-0.16	0.06	-0.13	0.09	1.00	2677	2895
age class combination – juvenile and juvenile	-0.40	0.10	-0.09	0.28	1.00	2534	3051
dyad ID (random effect)	0.20	0.04	0.12	0.26	1.01	544	783
multi-membership (random effect)	0.34	0.04	0.27	0.42	1.00	1240	2201
position (random effect)	0.23	0.09	0.12	0.44	1.00	1404	2182

Table S4 | Pairwise contrasts for *Model 2*: coordination (latency of arrival)

Pairwise contrast	*Median*	*Mean*	*Lower 95*	*Upper 95*	*Posterior Probability*
foraging associate - other site	3.96	3.99	0.40	7.65	98%
foraging associate - site resident	1.29	1.32	-1.28	4.00	83%
kin - foraging associate	-8.52	-8.62	-12.50	-5.23	0%
kin - neighbour	-7.18	-7.30	-12.11	-3.06	0.03%
kin - other site	-4.59	-4.63	-8.53	-0.85	0.8%
kin - site resident	-7.29	-7.31	-10.40	-4.26	0%
neighbour - foraging associate	-1.42	-1.32	-5.36	2.89	25.6%
neighbour - other site	2.62	2.67	-1.66	7.57	88.5%
neighbour - site resident	-0.09	-0.01	-3.60	3.96	48.1%
pair - foraging associate	-8.84	-8.91	-12.57	-5.69	0%
pair - kin	-0.28	-0.29	-3.66	2.94	43.1%
pair - neighbour	-7.51	-7.59	-12.27	-3.49	0%
pair - other site	-4.88	-4.9	-8.74	-1.42	0.3%
pair - site resident	-7.56	-7.60	-10.69	-4.72	0%
site resident - other site	2.70	2.67	-0.27	5.49	96.5%

Table S5 | Summary of *Model 3*: social tolerance (dyadic event duration)

Coefficient	*Est.*	*Est. Error*	*Lower 95% CrI*	*Upper 95% CrI*	*Rhat*	*Bulk ESS*	*Tail ESS*
Intercept	1.10	0.13	0.86	1.36	1.00	1256	1674
relationship – kin	0.64	0.12	0.41	0.88	1.00	2161	2877
relationship – neighbour	0.08	0.11	-0.13	0.29	1.00	2468	2295
relationship – other site	0.28	0.10	0.09	0.46	1.00	2285	2729
relationship – pair	1.22	0.12	1.00	1.46	1.00	1276	1702
relationship – site resident	0.27	0.07	0.13	0.41	1.00	1870	2212
arrival difference (z-transformed)	-0.03	0.02	-0.07	0.01	1.00	4044	2871
age class combination – adult and juvenile	0.54	0.08	0.38	0.69	1.00	2554	2867
age class combination – juvenile and juvenile	0.87	0.13	0.63	1.12	1.00	2812	2668
dyad ID (random effect)	0.52	0.03	0.45	0.58	1.01	918	1943
multi-membership (random effect)	0.54	0.05	0.44	0.65	1.00	1058	1607
position (random effect)	0.25	0.11	0.11	0.50	1.00	1197	1663

Table S6 | Pairwise contrasts for *Model 3*: social tolerance (dyadic event duration)

Pairwise contrast	*Median*	*Mean*	*Lower 95*	*Upper 95*	*Posterior Probability*
foraging associate - other site	-1.32	-1.34	-2.20	-0.59	0%
foraging associate - site resident	-1.65	-1.67	-2.33	-1.10	0%
kin - foraging associate	6.36	6.45	4.37	9.11	100%
kin - neighbour	5.79	5.89	3.79	8.43	100%
kin - other site	5.05	5.11	3.06	7.52	100%
kin - site resident	4.71	4.79	2.87	7.12	100%
neighbour - foraging associate	0.54	0.56	-0.14	1.39	93.7%
neighbour - other site	-0.77	-0.77	-1.76	0.20	6.7%
neighbour - site resident	-1.10	-1.10	-1.93	-0.27	0.5%
pair - foraging associate	7.25	7.37	5.07	10.45	100%
pair - kin	0.90	0.92	-1.89	3.88	74.6%
pair - neighbour	6.69	6.80	4.42	9.84	100%
pair - other site	5.92	6.03	3.71	9.00	100%
pair - site resident	5.59	5.70	3.44	8.61	100%
site resident - other site	0.33	0.33	-0.42	1.06	80.8%

Table S7 | Summary of *Model 3*: social tolerance (visit duration)

Coefficient	*Est.*	*Est. Error*	*Lower 95% CrI*	*Upper 95% CrI*	*Rhat*	*Bulk ESS*	*Tail ESS*
Intercept	3.43	0.06	3.31	3.56	1.00	1777	2080
relationship – kin	0.05	0.07	-0.09	0.18	1.00	3343	3101
relationship – neighbour	0.03	0.06	-0.09	0.15	1.00	3763	2937
relationship – other site	-0.02	0.06	-0.13	0.09	1.00	3251	2960
relationship – pair	0.13	0.05	0.03	0.24	1.00	3077	2578
relationship – site resident	0.03	0.04	-0.04	0.11	1.00	2530	2713
arrival difference (z-transformed)	0.52	0.01	0.49	0.55	1.00	4983	2590
age class combination – adult and juvenile	0.16	0.05	0.07	0.25	1.00	3661	2831
age class combination – juvenile and juvenile	0.30	0.07	0.16	0.45	1.00	3962	2853
dyad ID (random effect)	0.11	0.03	0.04	0.17	1.01	502	603
multi-membership (random effect)	0.21	0.03	0.15	0.27	10.00	1301	2277
position (random effect)	0.13	0.06	0.04	0.28	1.00	1140	1700

Table S8 | Summary of *Model 4*: displacement

Coefficient	*Est.*	*Est. Error*	*Lower 95% CrI*	*Upper 95% CrI*	*Rhat*	*Bulk ESS*	*Tail ESS*
Intercept	-1.57	0.19	-1.95	-1.20	1.00	2400	2615
relationship – other	0.77	0.17	0.45	1.11	1.00	4616	2823
relationship – solo	0.34	0.15	0.06	0.64	1.00	4523	3165
age class combination – adult, juvenile	-0.56	0.13	-0.82	-0.29	1.00	6004	3022
age class combination – juvenile, adult	0.33	0.15	0.03	0.62	1.00	5383	3254
age class combination – juvenile, juvenile	-0.66	0.30	-1.26	-0.08	1.00	7402	2931
dyad ID (random effect)	0.48	0.03	0.43	0.53	1.00	1331	2177
multi-membership (random effect)	0.77	0.06	0.66	0.89	1.00	1390	2281
position (random effect)	0.28	0.10	0.15	0.54	1.00	2032	2178

Table S9 | Summary of *Model 5:* queuing individual next visitor

Coefficient	*Est.*	*Est. Error*	*Lower 95% CrI*	*Upper 95% CrI*	*Rhat*	*Bulk ESS*	*Tail ESS*
Intercept	0.02	0.23	-0.43	0.48	1.00	6145	7317
relationship – kin	-0.42	0.21	-0.83	-0.02	1.00	12034	12471
relationship – neighbour	0.05	0.18	-0.31	0.41	1.00	12428	12948
relationship – other site	-0.37	0.17	-0.70	-0.03	1.00	10773	12168
relationship – pair	-0.74	0.18	-1.10	-0.38	1.00	9480	10846
relationship – site resident	-0.36	0.12	-0.60	-0.13	1.00	9237	11225
age class combination – adult and juvenile	-1.01	0.15	-1.31	-0.71	1.00	10931	12400
age class combination – juvenile and juvenile	-1.28	0.26	-1.81	-0.77	1.00	12127	12496
next visitor ID (random effect)	1.13	0.10	0.95	1.34	1.00	3564	7177
multi-membership (random effect)	1.35	0.13	1.11	1.61	1.00	4113	8465
dyad ID (random effect)	0.65	0.10	0.46	0.84	1.00	2104	3364
position (random effect)	0.43	0.22	0.14	0.97	1.00	4597	7199

Table S10 | Pairwise contrasts for *Model 5*: queueing individual next visitor

Pairwise contrast	*Median*	*Mean*	*Lower 95*	*Upper 95*	*Posterior Probability*
foraging associate - other site	0.11	0.11	0.03	0.19	99.6%
foraging associate - site resident	0.14	0.14	0.08	0.20	99.9%
kin - foraging associate	-0.27	-0.27	-0.36	-0.19	0%
kin - neighbour	-0.26	-0.26	-0.36	-0.15	0%
kin - other site	-0.16	-0.16	-0.26	-0.07	0.03%
kin - site resident	-0.14	-0.14	-0.20	-0.07	0.03
neighbour - foraging associate	-0.02	-0.02	-0.10	0.07	36%
neighbour - other site	0.09	0.09	-0.01	0.20	96.5%
neighbour - site resident	0.12	0.12	0.04	0.21	99.8%
pair - foraging associate	-0.18	-0.18	-0.26	-0.09	0%
pair - kin	0.10	0.10	0.01	0.20	98.4%
pair - neighbour	-0.16	-0.16	-0.26	-0.05	0.18%
pair - other site	-0.07	-0.07	-0.16	0.03	8.9%
pair - site resident	-0.04	-0.04	-0.11	0.04	16.4%
site resident - other site	-0.03	-0.03	-0.10	0.04	21.6%

Table S11 | Summary of *Model 6*: social learning (day of first visit)

Coefficient	*Estimate*	*Est. Error*	*Lower 95% CrI*	*Upper 95% CrI*	*Rhat*	*Bulk ESS*	*Tail ESS*
Intercept	5.28	0.01	5.26	5.31	1.00	1941	1766
independent (some)	-0.00	0.02	-0.04	0.03	1.00	5265	3146
followed	-0.06	0.01	-0.08	-0.03	1.00	4093	3187
day ringed (z-transformed)	0.00	0.01	-0.01	0.01	1.00	7216	2801
juvenile ID	0.01	0.00	0.00	0.02	1.00	1941	1766
position	0.03	0.01	0.01	0.06	1.00	1097	1455

Table S12 | Summary of *Model 7*: social learning (visit number)

Coefficient	*Est.*	*Est. Error*	*Lower 95% CrI*	*Upper 95% CrI*	*Rhat*	*Bulk ESS*	*Tail ESS*
Intercept	0.79	0.59	-0.40	1.94	1.00	2358	4311
independent (some)	0.16	0.27	-0.37	0.68	1.00	1334	2644
followed	0.72	0.26	0.19	1.23	1.00	1274	2570
day ringed (z-transformed)	-0.14	0.17	-0.47	0.20	1.01	979	2127
juvenile ID	1.95	0.15	1.68	2.27	1.00	1529	3468
position	1.48	0.45	0.85	2.59	1.00	4040	7368

References

1. Kings M. Foraging tactics and social networks in wild jackdaws [PhD Thesis]. University of Exeter; 2018.

2. Wickham H, Averick M, Bryan J, Chang W, McGowan L, François R, et al. Welcome to the Tidyverse. J Open Source Softw. 2019;4(43):1686. doi:10.21105/joss.01686

3. Wickham H, François R, Henry L, Müller K, Vaughan D. dplyr: A Grammar of Data Manipulation. 2023.

4. Grolemund G, Wickham H. Dates and times made easy with lubridate. J Stat Softw. 2011;40(3):1–25. doi:10.18637/jss.v040.i03

5. Gagolewski M. stringi: Fast and Portable Character String Processing in R. J Stat Softw. 2022;103(2). doi:10.18637/jss.v103.i02

6. Barrett T, Dowle M, Srinivasan A, Gorecki J, Chirico M, Hocking T, et al. data.table: Extension of \`data.frame\` [Internet]. 2025. Available from: https://cran.r-project.org/package=data.table

7. Franks DW, Ruxton GD, James R. Sampling animal association networks with the gambit of the group. Behav Ecol Sociobiol. 2010;493–503. doi:10.1007/s00265-009-0865-8

8. Psorakis I, Roberts SJ, Rezek I, Sheldon BC. Inferring social network structure in ecological systems from spatiotemporal data streams. J R Soc Interface. 2012;9(76):3055–66. doi:10.1098/rsif.2012.0223 PubMed PMID: 22696481.

9. Farine DR. Animal social network inference and permutations for ecologists in R using asnipe. Methods Ecol Evol. 2013;4(12):1187–94. doi:10.1111/2041-210X.12121

10. Cairns SJ, Schwager SJ. A comparison of association indices. Anim Behav. 1987;35(5):1454–69. doi:10.1016/S0003-3472(87)80018-0
